# Supplementary material for: Mapping the co-evolution of artificial intelligence, robotics, and the internet of things over 20 years (1998-2017)
Source: PLoS One. 2020 Dec 2;15(12):e0242984. doi: 10.1371/journal.pone.0242984 (PMC7710114; doi:10.1371/journal.pone.0242984)
Supplement: S1 Appendix — (PDF) [file pone.0242984.s001.pdf]

PLoS One S1 Appendix

Article title: Mapping the Co-Evolution of Artificial Intelligence, Robotics, and Internet of Things Over 20 Years (1998-2017)

Authors: Katy Börner, Leonard E. Cross, Michael Gallant, Shutian Ma, Adam S. Martin, Elizabeth Record, Olga B. Scrivner, Haici Yang, and Jonathan M. Dilger

The following Supporting Information is available for this article:

Supplementary text
Figures S1 to S3
Tables S1 to S6

Other supplementary materials for this manuscript include the following:

Code was made available at https://github.com/cns-iu/AICoEvolution

Data

Data used in this study comprises stakeholder needs and user study results, publication and funding data as well as keyword dictionaries and other data files needed for keyword extraction.

Stakeholder Needs and User studies include the Qualtrics survey materials (6 demographic questions, 24 questions with 5 visualizations) results available at https://github.com/cns-iu/AICoEvolution. Participants fell into two age groups: 31-40 age group (2) and 51-60 age group (3). One participant was female and 4 were male. All five were native English speakers.

Publication data was retrieved from the IUNI Web of Science (WoS) Data Enclave (Indiana University Network Science Institute, 2018) and Clarivate Web-of-Science Portal. There exist 66,036,893 publications for the period 2010-2017. Exactly 32,716 publications are extracted with 291 publications overlapping.

Funding data was retrieved from the NSF Award Search Portal NSF portal - https://nsf.gov/awardsearch and downloaded in bulk from https://nsf.gov/awardsearch/download.jsp

Dictionaries

Other datasets include a stopword list from the Stanford natural language processing (NLP) library (Pan, Yan, Wang, & Hua, 2015), extracted WoS keywords related to AI, robotics, and IoT, the UCSD Map of Science and Classification System (Börner et al., 2012) available at

https://cns.iu.edu/2012-UCSDMap.html and the list of abbreviation used to describe funding agencies and organizations on WoS portal available at https://github.com/cns-iu/AICoEvolution.

Code

Software used in this study includes term entity extraction algorithms to identify keywords in funding titles and descriptions; algorithms to compute term frequency bursts, and network layout algorithms and tools such as Gephi, Make-a-Viz, and OpenRefine.

Term Extraction

MaxMatch algorithm (Wong & Chan, 1996) was used to extract keywords from publication and funding data. For more details see https://github.com/cns-iu/cjobs (Börner et al., 2018).

Methods

Burst Detection and Visualization. Kleinberg's (2002) burst detection algorithm is used to detect sudden increases in how often certain keywords are used in temporal data streams.

Co-Author Networks. The Sci2 Tool was used to extract a co-author network using the co-author column.

Network Layout Algorithms. Gephi (Version 0.9.2) (Bastian, Heymann, & Jacomy, 2009) was used to compute data overlays with the following plugins:

- **ForceAtlas2** was used to layout the *Co-Author Network* figure.
- **GeoLayout** was used to create 1) the *Co-Author network overlaid on US map* with mercator basemap and the *Temporal Convergence* figure using latitude and longitude coordinates to display nodes.

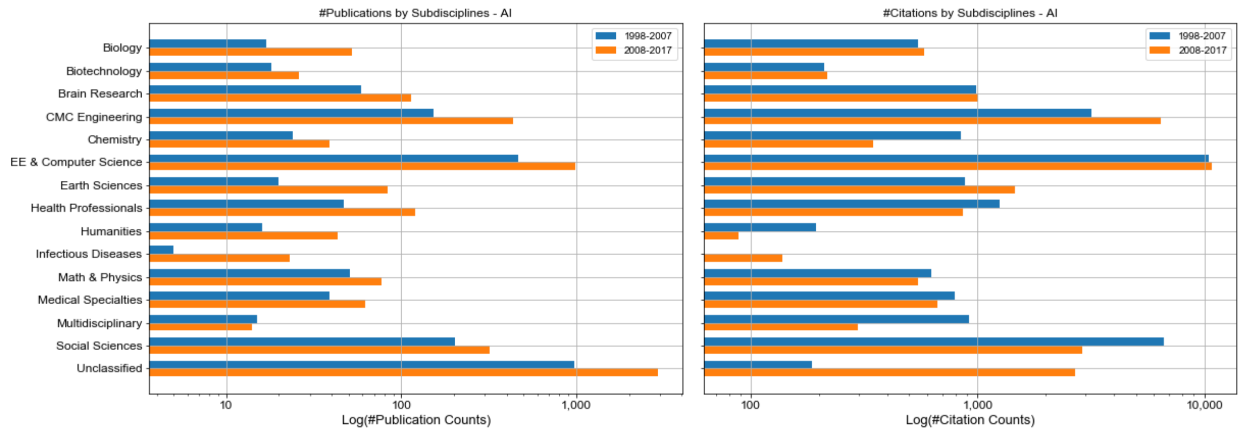

Fig S1. Topical coverage of AI publications published in 1998-2007 and 2008-2017 in terms of number of papers (left) and number of citations (right). Abbreviations used: CMC – Chemical, Mechanical, and Civil; EE – Electrical Engineering).

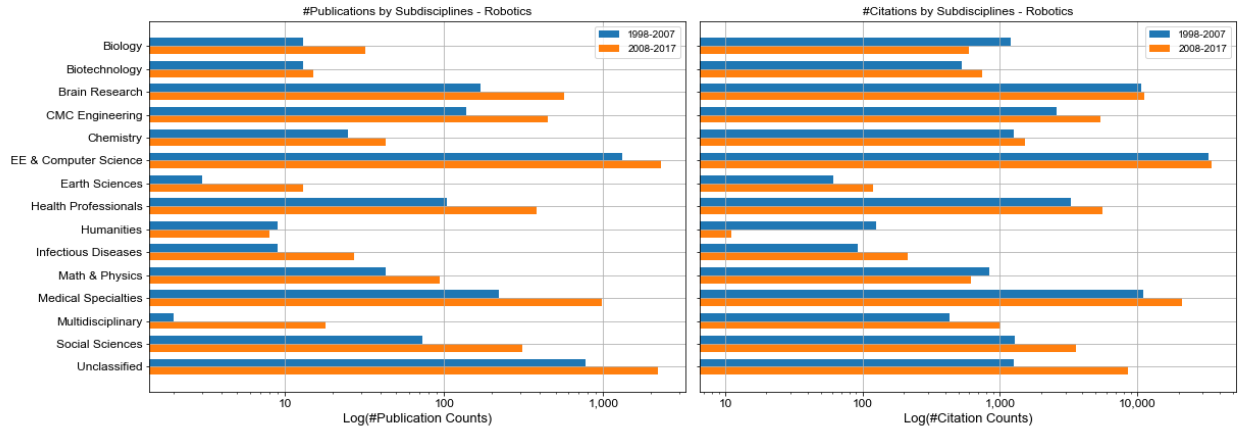

Fig S2. Topical coverage of Robotics publications published in 1998-2007 and 2008-2017 in terms of number of papers (left) and number of citations (right).

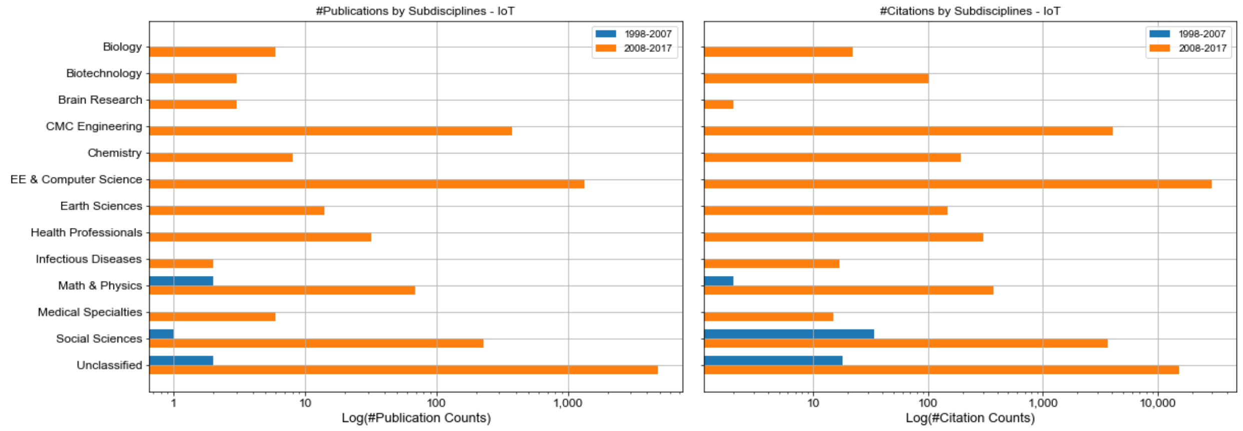

Fig S3. Topical coverage of IoT publications published in 1998-2007 and 2008-2017 in terms of number of papers (left) and number of citations (right).

Table S1. **Table Summary for Fig S1**. Topical coverage of AI publications published in 1998-2007 and 2008-2017 in terms of number of papers and number of citations. Unclassified labels and NULL labels were combined into Unclassified discipline.

	1998-2007		2008-2017			
**Disciplines**	**#Pubs**	**#Citation**	**#Pubs**	**#Citation**	**#Total Pubs**	**#Total Citations**
Biology	17	549	52	584	69	1133
Biotechnology	18	211	26	218	44	429
Brain Research	59	990	114	1005	173	1995
Chemical, Mechanical, & Civil Engineering	152	3182	436	6432	588	9614
Chemistry	24	847	39	345	63	1192
Earth Sciences	20	876	84	1469	104	2345

Electrical Engineering & Computer Science	462	10480	982	10853	1444	21333
Health Professionals	47	1251	120	865	167	2116
Humanities	16	194	43	88	59	282
Infectious Diseases	5	58	23	138	28	196
Math & Physics	51	623	77	548	128	1171
Medical Specialties	39	795	62	668	101	1463
Multidisciplinary	15	913	14	296	29	1209
Social Sciences	203	6661	318	2883	521	9544
Unclassified	974	185	2922	2696	3896	2881
Total	2102	27815	5312	29088	7414	56903

Table S2. **Table Summary for Fig S2**. Topical coverage of Robotics publications published in 1998-2007 and 2008-2017 in terms of number of papers and number of citations. Unclassified labels and NULL labels were combined into Unclassified discipline.

	1998-2007		2008-2017			
**Disciplines**	**#Pubs**	**#Citations**	**#Pubs**	**#Citations**	**#Total Pubs**	**#Total Citations**
Biology	13	1206	32	594	45	1800
Biotechnology	13	527	15	746	28	1273
Brain Research	169	10772	569	11188	738	21960
Chemical, Mechanical, & Civil Engineering	138	2605	445	5376	583	7981
Chemistry	25	1272	43	1531	68	2803
Earth Sciences	3	61	13	120	16	181
Electrical Engineering & Computer Science	1310	33083	2311	35043	3621	68126
Health Professionals	104	3281	381	5563	485	8844
Humanities	9	125	8	11	17	136
Infectious Diseases	9	93	27	215	36	308
Math & Physics	43	841	94	614	137	1455
Medical Specialties	221	11028	981	21181	1202	32209
Multidisciplinary	2	431	18	1016	20	1447
Social Sciences	73	1281	308	3581	381	4862
Unclassified	1689	1262	4865	8518	6554	9780
Total	3821	67868	10110	95297	13931	163165

Table S3. **Table Summary for Fig S3**. Topical coverage of IoT publications published in 1998-2007 and 2008-2017 in terms of number of papers and number of citations. Unclassified labels and NULL labels were combined into Unclassified discipline.

1998-2007	2008-2017

Disciplines	#Pubs	#Citations	#Pubs	#Citations	#Total Pubs	#Total Citations
Biology	0	0	6	22	6	22
Biotechnology	0	0	3	103	3	103
Brain Research	0	0	3	2	3	2
Chemical, Mechanical, & Civil Engineering	0	0	377	4110	377	4110
Chemistry	0	0	8	194	8	194
Earth Sciences	0	0	14	147	14	147
Electrical Engineering & Computer Science	0	0	1338	29997	1338	29997
Health Professionals	0	0	32	302	32	302
Infectious Diseases	0	0	2	17	2	17
Math & Physics	2	2	69	374	71	376
Medical Specialties	0	0	6	15	6	15
Social Sciences	1	34	226	3665	227	3699
Unclassified	4	18	9280	15469	9284	15487
Total	7	54	11364	54417	11371	54471

Table S4. Top-5 research articles by #Citations.

Title	Year	#Citation
**AI**		
Artificial neural networks (the multilayer perceptron) - A review of …	1998	681
Psychological aspects of natural language use: Our words, our …	2003	735
Advances in Diagnostic Techniques for Induction Machines	2008	559
ViBe: A Universal Background Subtraction Algorithm for Video Sequ...	2011	702
A review on the prediction of building energy consumption	2012	440
**Robotics**		
Vision for mobile robot navigation: A survey	2002	609
An inexpensive, automation-friendly protocol for recovering high-qual…	2006	705
Effects of robot-assisted therapy on upper limb recovery after stroke...	2008	682
A survey of robot learning from demonstration	2009	721
A review of shape memory alloy research, applications and opportunities	2014	700
**IoT**		
The Internet of Things: A survey	2010	3,859
Internet of Things (IoT): A vision, architectural elements, and future…	2013	2,344
Recommender systems survey	2013	693
Internet of Things in Industries: A Survey	2014	733
Internet of Things: A Survey on Enabling Technologies, Protocols...	2015	902

Note. Data Citation (top 5 publications sorted by Citation) is available in data section: https://github.com/cns-iu/AICoEvolution

Table S5. Top 10 Bursts for each domain in Publications and NSF Awards

Publications	Burst Strength	NSF Awards	Burst Strength
**AI**			
Learning (Artificial intelligence)	39.29	Machine learning	13.04
Machine learning	26.41	Education	9.96
Neural networks	25.61	Making	9.20
Distributed artificial intelligence	20.47	Big data	8.97
Deep learning	18.49	Building	8.06
Expert systems	17.99	Web	7.85
Big data	16.17	Interaction	7.29
Genetic algorithms	11.12	Society	7.06
Feature extraction	10.58	Deep learning	7.05
Internet of things	9.35	Data science	7.01
**Robotics**			
Soft robotics	58.15	Law	31.55
Cloud robotics	23.38	Recovery	27.84
Educational robotics	19.49	Impacts	21.00
Control technology	15.33	STEM	19.91
Social robotics	14.29	Algebraic geometry	17.24
3d printing	14.18	School	14.35
Teleoperation	13.98	College	13.86
Marine robotics	13.05	Soft robotics	13.70
Welding	12.96	Machine learning	13.56
Laparoscopy	12.87	Biology	12.60
**IoT**			
RFID	62.16	Vehicles	4.26
The internet of things	33.63	Monitor	4.16
Radio frequency identification	15.07	Computation	4.15
6Lowpan	13.20	Runtime	3.47
EPC	13.18	Integrated circuits	3.47
Future internet	12.91	Experiments	3.32
Fog computing	12.06	Robust	3.30
Edge computing	10.38	Community	3.27
Lora	10.13	Social	3.01
Lorawan	10.01	Vehicle	2.98

Table S6. The NSF Funding/Organizations Summary for AI, robotics, and IoT by countries.

Unique Name	Name Variations	#Paper	Country	Type
CAPES	CAPES	42	Brazil	Funding
CNPQ	CNPQ;THE NATIONAL COUNCIL FOR SCIENTIFIC AND	56	Brazil	Funding

	TECHNOLOGICAL DEVELOPMENT			
CONACYT	CONACYT	38	Mexico	Funding
CPSF	CHINA POSTDOCTORAL SCIENCE FOUNDATION	64	China	Funding
DARPA	DEFENSE ADVANCED RESEARCH PROJECTS AGENCY	73	USA	Funding
EC	EUROPEAN COMMISSION	335	Europe	Funding
Common Mkt	EUROPEAN COMMUNITY	97	Europe	Funding
EPSRC	ENGINEERING AND PHYSICAL SCIENCES RESEARCH COUNCIL	411	UK	Funding
EU	EUROPEAN UNION	539	Europe	Funding
FAPESP	SÃO PAULO RESEARCH FOUNDATION	27	Brazil	Funding
FRF	FUNDAMENTAL RESEARCH FUNDS FOR THE CENTRAL UNIVERSITIES	264	China	Funding
JSPS	JSPS KAKENHI	37	Japan	Funding
MICINN	SPANISH MINISTRY OF SCIENCE AND INNOVATION	34	Spain	Funding
MSTT	MINISTRY OF SCIENCE AND TECHNOLOGY TAIWAN	40	China	Funding
NIH	NIH;NATIONAL INSTITUTES OF HEALTH	214	USA	Funding
NSF	NATIONAL SCIENCE FOUNDATION;NSF	1350	USA	Funding
NSFC	NATIONAL NATURAL SCIENCE FOUNDATION OF CHINA; NATIONAL SCIENCE FOUNDATION OF CHINA	1890	China	Funding
NSFK	BASIC SCIENCE RESEARCH PROGRAM THROUGH THE NATIONAL RESEARCH FOUNDATION OF KOREA NRF MINISTRY OF EDUCATION	56	Korea	Funding
ONR	OFFICE OF NAVAL RESEARCH;ONR	151	USA	Funding
SNSF	SWISS NATIONAL SCIENCE FOUNDATION	69	SWISS	Funding
BUPT	BEIJING UNIVERSITY OF POSTS TELECOMMUNICATIONS	262	China	Organization
CAS	CHINESE ACADEMY OF SCIENCE	500	China	Organization
CMU	CARNEGIE MELLON UNIVERSITY	542	USA	Organization
CNRS	CENTRE NATIONAL DE LA RECHERCHE SCIENTIFIQUE CNRS	1298	France	Organization

DoD	UNITED STATES DEPARTMENT OF DEFENSE	319	USA	Organization
Helmholtz	HELMHOLTZ ASSOCIATION	402	Germany	Organization
IAU	ISLAMIC AZAD UNIVERSITY	175	UAE	Organization
IIT	INDIAN INSTITUTE OF TECHNOLOGY SYSTEM IIT SYSTEM; INDIANA INSTITUTE OF TECHNOLOGY SYSTEM	280	India	Organization
INRIA	INRIA	117	France	Organization
MIT	MASSACHUSETTS INSTITUTE OF TECHNOLOGY MIT	480	USA	Organization
PolyU	HONG KONG POLYTECHNIC UNIVERSITY	132	China	Organization
PSHE	PENNSYLVANIA COMMONWEALTH SYSTEM OF HIGHER EDUCATION PCSHE	140	USA	Organization
S. Anna	SCUOLA SUPERIORE SANT ANNA	313	Italy	Organization
Saclay	UNIVERSITY PARIS SACLAY COMUE	145	France	Organization
SUSF	STATE UNIVERSITY SYSTEM OF FLORIDA	149	US	Organization
UCA	UNIVERSITE COTE D AZUR COMUE	1214	France	Organization
UC System	UNIVERSITY OF CALIFORNIA SYSTEM	1050	US	Organization
U London	UNIVERSITY OF LONDON	159	UK	Organization
USG	UNIVERSITY SYSTEM OF GEORGIA	504	US	Organization
Utsinghua	TSINGHUA UNIVERSITY	138	China	Organization
UT System	UNIVERSITY OF TEXAS SYSTEM	316	US	Organization

References

Bastian, M., Heymann, S., & Jacomy, M. (2009). Gephi: an open source software for exploring and manipulating networks. In E. Adar, M. Hurst, T. Finin, N. Glance, N. Nicolov, & B. Tseng (Eds.), *Proceedings of the Third International Conference on Weblogs and Social Media* (pp. 361–362). Menlo Park, California: The AAAI Press. Retrieved from https://gephi.org/users/publications/

Börner, K., Klavans, R., Patek, M., Zoss, A. M., Biberstine, J. R., Light, R. P., … Boyack, K. W. (2012). Design and update of a classification system: The UCSD map of science. *PLoS ONE*, *7*(7), e39464. https://doi.org/10.1371/journal.pone.0039464

Börner, K., Scrivner, O., Gallant, M., Ma, S., Liu, X., Chewning, K., … Evans, J. A. (2018). Skill discrepancies between research, education, and jobs reveal the critical need to supply soft skills for the data economy. *Proceedings of the National Academy of Sciences of the United States of America*, *115*(50), 12630–12637. https://doi.org/10.1073/pnas.1804247115

Indiana University Network Science Institute. (2018). Web of Science (WoS). Retrieved July 13, 2018, from http://iuni.iu.edu/resources/web-of-science

Kleinberg, J. (2002). Bursty and hierarchical structure in streams. In D. Hand, D. A. Keim, & R.

NG (Eds.), *Proceedings of the Eighth ACM SIGKDD International Conference on Knowledge Discovery and Data Mining - KDD '02* (pp. 91–101). New York, New York, USA: ACM Press. https://doi.org/10.1145/775047.775061

Pan, X., Yan, E., Wang, Q., & Hua, W. (2015). Assessing the impact of software on science: A bootstrapped learning of software entities in full-text papers. *Journal of Informetrics*, *9*(4), 860–871. https://doi.org/10.1016/J.JOI.2015.07.012

Sci2 Team. (2009). Science of Science (Sci2) Tool. Indiana University and SciTech Strategies. Retrieved from https://sci2.cns.iu.edu

Wong, P.-K., & Chan, C. (1996). Chinese word segmentation based on maximum matching and word binding force. *Proceedings of the 16th Conference on Computational Linguistics*, *1*, 200–203. Retrieved from http://citeseerx.ist.psu.edu/viewdoc/download?doi=10.1.1.14.3112&rep=rep1&type=pdf
