# Supplementary material for: Mapping the co-evolution of artificial intelligence, robotics, and the internet of things over 20 years (1998-2017)
Source: PLoS One. 2020 Dec 2;15(12):e0242984. doi: 10.1371/journal.pone.0242984 (PMC7710114; doi:10.1371/journal.pone.0242984)
Supplement: Attachment — Submitted filename: Response to Reviewers.pdf [file pone.0242984.s003.pdf]

Dear Editor:

Thank you for the detailed review of our manuscript on *Mapping the co-evolution of artificial intelligence, robotics, and the internet of things over 20 years (1998-2017).*

We submit here a revised version of our paper, which implements suggestions by Reviewer 2. We also include a point-by-point response to each of the comments.

Thank you again for your continued consideration of our work. We hope this revision addresses all remaining concerns and merits publication, but we stand ready to make whatever adjustments you deem necessary.

In response to comments from Reviewer 2:

1. While I agree with the authors' argument that the work examines areas of strategic interest, my concern is that these selected areas happen to be very correlated with each other. This raises the question of how useful the presented approach would be when the selected areas are not correlated with each other. For example, if a policy maker wanted to make inferences about say, 'Power and energy management' and 'robotics' (or any two fields that don't seem to be immediately/obviously correlated), I wonder how the approach would fare. My point is that currently, the authors show how their approach can be useful when related fields are considered. While this is useful, I think it is also equally important to show what the results would look like when your approach is used to evaluate seemingly unrelated fields of research.

Response

Thank you for sharing your concern. Many strategic decision makers (research team leads, funders, teachers) are very interested to understand the interplay of research areas that co-evolve and impact each other's growth. The paper presents general workflows and novel visualizations and exemplifies them in an exemplary analysis of three research areas that have high R&D impact and value and that were identified by leading decision makers.

Please do note that the same workflows can be used to understand the evolution of unrelated research areas. For example, data on 'Power and energy management' and 'robotics' can be compiled via the Web of Science and NSF award portals using relevant query terms. The NLP MaxMatch algorithm for keywords extraction and other workflows on Github https://github.com/cns-iu/AICoEvolution/ can be used to analyze and visualize these research areas. Even if the research areas are unrelated and no matching terms are found (aka null

convergence), emerging topical areas in published work and awards, bursts, and co-author networks can be studied. However, this is not the main focus of the present paper.

2. I believe replicating at least some of the experiments on other non-correlated fields would serve to strengthen the contribution of this paper, which is also a concern raised by Reviewer 1. Otherwise, it would seem like the authors only considered the convenient choice of correlated fields of research.

Response

Just to reiterate, the paper introduces two novel visualizations (co-bursts and convergence) that are particularly valuable for studying the convergence of research areas. Both visualizations were exemplarily used to map three research areas of strategic importance and both were evaluated by domain experts.

As we write in our original response to Reviewer 2, "the work presented here is more pragmatic--it starts with a set of research areas that are of strategic interest to different governmental labs as well as industry representatives so that study results can directly support data-driven decision making."

Reviewer 1 was originally concerned about the "lack of significant results and major findings" and not the choice of the research areas. In our recent revision, we pointed out that the paper introduces two novel visualizations (co-bursts and convergence) that were evaluated by domain experts which fully addressed the concerns of Reviewer 1.

3. The authors' response to point 8. from the first round of reviews did not really address my concern. I am not suggesting the analysis be re-done based on the current trend (which could be quantified by say, the rate of change in the citations and publications), but I think the authors need to justify why using the total citations and publications is more relevant than using the current trends of these quantities, when it comes to the strategic interests of governments and industries. At the very least, the authors should acknowledge that other measures (other than the total pubs.+cites.) could be also be used.

Response

Thank you for clarifying your concern. In our approach, we used a variety of metrics in addition to #publications and #citations, such as publication sources (journals), disciplines and subdisciplines, authors and their co-authorship relations, authors' geographical locations, keywords, extracted terms (NLP MaxMatch feature engineering method), award $ amounts, type of organization and funding agency.

We added the following text (p.2): "Other recent studies introduced several novel NLP methods to measure research diversity and interdisciplinarity. For example, topic modeling has been used to measure the degree of topic diversity (Yegros-Yegros et al., 2015), Shannon's entropy measure was applied to compute technological diversity using EU-funded nanotechnology projects data (Paez-Aviles et al., 2018). This paper uses robust and widely used topic identification methods and focuses on visualizing the emergence, co-evolution, and convergence of science and technology areas."

4. The point about changes in terminology is an important one (comment 11. from the first round of reviews). I hope the authors include the points mentioned in their response, to the main manuscript.

Response

Thank you for suggesting this. We added to the study limitation (p.15): "Finally, we focused only on a small, static subset of keywords and their alternatives (IoT vs Internet of Things) found in abstracts, titles, or keywords and did not examine contextual or semantic changes of terms over time."

5. The text overlaid on top of the graph in Fig 9 can be removed (point 33. from 1st round of reviews). This could make the figure significantly less cluttered, without any loss of information, as the corresponding color codes are already mentioned in the legend.

Response

The visualization was computed using the Make-A-Vis tool. We prefer to keep the current figure to support easy and complete reproducibility of the presented workflow. Labeling major areas of science in a map of all sciences is analogous to providing names of major geographic areas, e.g., continents or countries, on a map of the world.

6. Regarding point 36., I still think it would be valuable to add a section on the limitations and future issues that remain to be addressed. This could be useful for those aiming to develop similar visualization tools in the future.

Response

Thank you for this suggestion. We added the following text to (p.15): "The presented research has several limitations. First, the analysis uses two high-quality, high-coverage data sources (Web of Science and NSF Awards database). But other data (e.g., publication data from the

ArXiv preprint repository (Klinger et al., 2020) or patents to capture technology evolution (Paez-Aviles et al., 2018) could be added in future studies to capture science and technology developments. Note that an inclusion of additional data sources would require disambiguation and cleaning of author names and geographical locations. While Web of Science provides information on paper citations, ArXiv does not. Going forward, we are interested to explore additional datasets such as Federal Business Opportunities (FBO) data that would make it possible to gain a more comprehensive understanding of the S&T funding landscape. Secondly, we used a variety of metrics such as the number of publications and citations, publication sources (journals), disciplines and subdisciplines, authors and their co-authorship relations, authors' geographical locations, keywords, extracted terms (NLP MaxMatch feature engineering method), award $ amounts, type of organization and funding agency. Future work might also like to consider additional metrics such as authors' diversity proposed by Paez-Aviles et al. (2018) and topic diversity recently suggested by Klinger et al. (2020). Finally, we focused on a small, static subset of keywords and their alternatives (IoT vs Internet of Things) found in abstracts, titles, or keywords and did not examine contextual or semantic changes of terms over time."

References

Klinger, J., Mateos-Garcia, J., & Stathoulopoulos, K. (2020). A narrowing of AI research. *ArXiv*: 2009.10385. Retrieved from https://arxiv.org/abs/2009.10385

Páez-Avilés, C., Van Rijnsoever, F. J., Juanola-Feliu, E., & Samitier, J. (2018). Multi-disciplinarity breeds diversity: the influence of innovation project characteristics on diversity creation in nanotechnology. *Journal of Technology Transfer*, *43*(2), 458–481. https://doi.org/10.1007/s10961-016-9553-9

Kind Regards,

Olga Scrivner

Olga Scrivner
